# Predictive factors for early progression during induction chemotherapy and chemotherapy-free interval: analysis from PRODIGE 9 trial

**DOI:** 10.1038/s41416-020-0735-8

**Published:** 2020-02-04

**Authors:** Thomas Aparicio, Jaafar Bennouna, Karine Le Malicot, Valérie Boige, Julien Taieb, Olivier Bouché, Jean-Marc Phelip, Eric François, Christian Borel, Roger Faroux, Laetitia Dahan, Jean-Baptiste Bachet, Joelle Egreteau, Marie-Christine Kaminsky, Jean-Marc Gornet, Oana Cojocarasu, Mohamed Gasmi, Véronique Guerin-Meyer, Côme Lepage, François Ghiringhelli, for PRODIGE investigators/collaborators

**Affiliations:** 1 Gastroenterology and Digestive Oncology Department, Hôpital Saint Louis, APHP.Nord, Université de Paris, Paris, France; 2 Gastroenterology and Digestive Oncology Department, IMAD, Nantes University Hospital, Nantes, France; 3 Fédération Francophone de Cancérologie Digestive (FFCD); Statistics Department, EPICAD INSERM LNC-UMR 1231, Université de Bourgogne et Franche Comté, Dijon, France; 4 Medical Oncology Department, Gustave Roussy, Villejuif, France; 5 Hepato-Gastroenterology and GI Oncology Department, Hôpital Européen Georges Pompidou, APHP, Université de Paris, Paris, France; 6 Hepato-Gastroenterology Department, University Hospital Robert Debré, Reims, France; 7 Hepato-Gastroenterology Department, Saint Etienne University Hospital, Hôpital Nord, Saint Priest en Jarez, France; 8 Oncology Department, Antoine Lacassagne Center, Nice, France; 9 Oncology Department, Paul Strauss Center, Strasbourg, France; 10 Hepato-Gastroenterology Department, Hospital Les Oudairies, La Roche sur Yon, France; 11 Hepato-Gastroenterology and Oncology Department, University Hospital la Timone, Marseille, France; 12 Hepato-Gastroenterology Department, Hôpital Pitié-Salpêtrière, APHP, Sorbonne Université, Paris, France; 13 Radiotherapy and Oncology Department, Centre Hospitalier Bretagne Sud, Lorient, France; 14 Oncology Department, Institut de Cancérologie de Lorraine, Vandoeuvre-lès-Nancy, France; 15 Onco-Hematology Department, Centre hospitalier du Mans, Le Mans, France; 16 Hepato-Gastroenterology Department, Hôpital Nord, Marseille, France; 17 Radiotherapy and Oncology Department, ICO Site Paul Papin, Angers, France; 18 Hepato-Gastroenterology Department, University Hospital Le Bocage, EPICAD INSERM LNC-UMR 1231, Université de Bourgogne et Franche Comté, Dijon, France; 19 Oncology Department, Centre Georges-François Leclerc, Dijon, France

**Keywords:** Colorectal cancer, Prognostic markers

## Abstract

**Background:**

Identifying patients with metastatic colorectal cancer who will have an early disease progression during induction chemotherapy (IC) and identifying patients who may have a chemotherapy-free interval (CFI) after IC are two major challenges.

**Methods:**

A logistic model was used to identify factors associated with early progression during IC and with short duration of the first CFI in 488 patients enrolled in the PRODIGE 9 trial. Independent factors were defined with a threshold 0.10.

**Results:**

In multivariate analysis, baseline leukocytes >10 × 10^9^/L (OR = 1.98 [1.02–3.8], *p* = 0.04), and stable or increasing CEA at 2 months (OR = 3.61 [1.68–7.75], *p* = 0.01) were independent factors associated with progression during IC. Male gender (OR = 1.725 [0.92–3.325], *p* = 0.09) and no tumour response at first evaluation (OR = 1.90 [0.96–3.76], *p* = 0.07) were significantly associated with a short CFI. The presence of *BRAF* V600E mutation was also associated with short CFI (OR = 4.59 [0.95; 22.3], *p* = 0.058).

**Conclusion:**

High baseline leukocyte count and the lack of CEA decrease level at first evaluation were associated with early progression, and could be in favour of early chemotherapy intensification. Male gender, no tumour response at first evaluation and *BRAF* mutation are associated with a short CFI, and may be considered for maintenance chemotherapy after IC.

**Clinical trial number:**

NCT00952029.

## Background

The prognosis of patients with metastatic colorectal cancer (mCRC) has been significantly improved by the use of several consecutive chemotherapy drugs.^1^ First-line chemotherapy irinotecan, 5-fluorouracil (5FU) and bevacizumab became a standard of care in mCRC.^2,3^ Due to the prolonged survival, up to 2–3 years under treatment, and in order to avoid heavy treatment burden and toxicity, chemotherapy-free intervals (CFI) were proposed in different studies with oxaliplatin- or irinotecan-based first-line induction chemotherapy (IC).^4–6^

A pooled analysis of several trials has shown that CFI did not impair overall survival (OS) and advocate for biomarker research to define a predictive factor.^7^ Nevertheless, the predictive factors associated with a long duration of CFI remain poorly studied. On the other hand, early identification of patients who will have an early progression during IC is an important challenge in order to intensify front-line treatment. Previous recent large trials have reported prognostic factors for progression-free survival (PFS),^8–10^ but not for early progression within the first 6 months of treatment. Moreover, two of these trials evaluated front-line treatment in the subgroup of patients with wild-type *RAS* mCRC.^8,9^ The randomised phase 3, PRODIGE 9 study, aimed to assess the tumour control duration with bevacizumab maintenance or observation after irinotecan-based IC combined with bevacizumab.^11^ Two other recent trials randomised patients without progression after IC.^12,13^ As the randomisation was performed prior to the front-line treatment whatever the *RAS* status, analysis of the PRODIGE 9 trial allows the determination of prognostic factors in all patients with mCRC.

The purpose of this ancillary study of the PRODIGE 9 trial is to determine the prognostic factors for early progression during IC, and during the first CFI in the subgroup of patients without progression of the disease after IC.

## Methods

PRODIGE 9 was an open-label, randomised, multicentre, phase 3 study conducted by the Fédération Francophone de Cancérologie Digestive (FFCD) and the PRODIGE intergroup in 66 French centres comparing IC with FOLFIRI plus bevacizumab followed by bevacizumab monotherapy (maintenance arm) or the same induction treatment followed by observation.^11^ The IC was planned for 12 cycles (6 months) after randomisation. The main eligible criteria were histologically proven, non-resectable mCRC, WHO status ≤ 2, life expectancy ≥ 3 months, absence of previous chemotherapy or anti-angiogenic therapy for metastatic disease. The primary endpoint was the tumour control duration defined as the time elapsed between randomisation and tumour progression during a chemotherapy sequence. There were no significant differences between the two arms not only for the primary endpoint but also for the median duration of the first CFI.^14^

Progression or death during IC was considered as the event for prognostic factor analysis of early progression during IC. Only patients without progression during induction chemotherapy who have entered in the CFI phase were analysed for the determination of prognostic factors related to the duration of the first CFI. Duration of the first CFI was defined as the time between the end of IC and the first reintroduction of chemotherapy whatever the regimen or death.

The following factors were evaluated for early progression during IC, and for early (<3 months) or late progression (>5 months) during the first CFI: treatment arm, sex, age, WHO performance status (PS), resection of primary tumour, number of metastatic sites, primary localisation, leukocytes, platelets, alkaline phosphatase, carcinoembryonic antigen (CEA) level, tumour *KRAS* status, tumour *BRAF* status (tumour with *KRAS* mutation was considered as *BRAF* wild type) and decrease in CEA >50% at 2 months. Tumour response at the end of induction chemotherapy and early shrinkage at first evaluation were evaluated only for CFI duration. A logistic model was used to identify the prognostic factors. A significance level of 0.2 was required to enter into the final univariate model and to stay in the multivariate model. We have considered as interesting a factor with a level of 0.10 in the multivariate model.

## Results

Between March 2010 and July 2013, the PRODIGE 9 trial enrolled 494 patients. Among them, six patients withdrew their consent (3) or were never treated (3); thus, the modified intent-to-treat population was 488 patients randomly assigned to either FOLFIRI plus bevacizumab IC followed by bevacizumab maintenance (*n* = 245), or to the same IC followed by observation during CFI (*n* = 243).

**Fig. 1:**
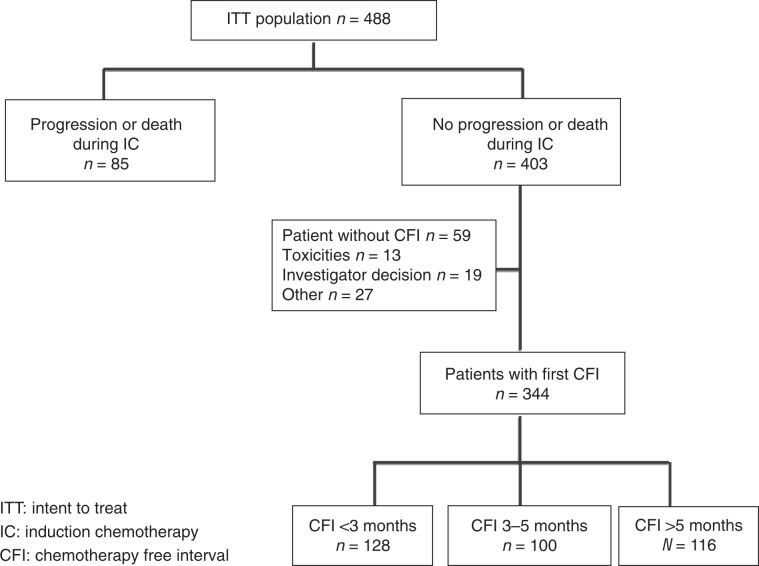
Flowchart.

Disease progression or death during IC occurred in 85 (17.4%) patients. Among the 403 patients who have no progression or death during IC, 59 had no CFI due to investigator decision, toxicities or other reasons. Among the remaining 344 patients, 128 (37.2%) patients had a CFI <3 months, 100 (29%) patients had a CFI between 3 and 5 months and 116 (33.7%) patients had a CFI >5 months (Fig. 1).

### Factors associated with progression during induction chemotherapy

Baseline characteristics of patients with and without tumour progression during IC are presented in Supplementary Table S1. Univariate analysis revealed that baseline WHO performance status of 2, baseline leukocytes >10 × 10^9^/L, baseline CEA upper limit of normal and stable or increasing CEA at 2 months after the beginning of IC were associated with a higher risk of progression during IC (Table 1). In multivariate analysis, baseline leukocytes >10 × 10^9^/L and stable or increasing CEA at 2 months were independent factors associated with progression during IC (Table 2). The ratio of neutrophils/leukocytes was also explored, but adds no additional result to the leukocyte count alone (data not shown).

**Table 1:** Univariate analysis of characteristics associated with a progression during induction chemotherapy.

Characteristics		OR for progression	[95% CI], *p* value
Gender	Female vs male	1.28	[0.79–2.07], *p* = 0.31
Age	≤65 vs >65	1.16	[0.73–1.86], *p* = 0.52
WHO performance status	1 vs 0 2 vs 0	1.59 3.79	[0.95–2.65], *p* = 0.43 [1.78–8.09], *p* = 0.002
Primary tumour resected	No vs yes	1.26	[0.79–2.02], *p* = 0.33
Number of metastatic sites	>1 vs 1	1.16	[0.71–1.88], *p* = 0.55
Primary location	Right colon vs left colon or rectum	0.87	[0.62–1.31], *p* = 0.41
Baseline leukocytes	>10 × 10^9^/L vs ≤10 × 10^9^/L	1.91	[1.16–3.15], *p* = 0.01
Baseline platelet	≥400 × 10^9^/L vs <400 × 10^9^/L	1.68	[0.92–3.08], *p* = 0.09
Baseline alkaline phosphatase	>300 vs ≤300 U/L	1.39	[0.79–2.44], *p* = 0.25
Baseline CEA	>ULN vs normal	2.46	[0.86–7.09], *p* = 0.10
Two months CEA vs baseline CEA	Stable or increase vs decrease >50%	3.00	[1.44–6.23], *p* = 0.01
Tumour *KRAS*	Mutated vs wild type	1.12	[0.65–1.92], *p* = 0.70
Tumour *BRAF*	Mutated vs wild type	1.30	[0.42–4.04], *p* = 0.65

*CEA* carcinoembryonic antigen, *ULN* upper limit of normal.

**Table 2:** Multivariate analysis of characteristics associated with a progression during induction chemotherapy.

Characteristics	*N* = 363	OR for progression	[95% CI], *p* value
WHO performance status	1 vs 0 2 vs 0	1.18 1.65	[0.61–2.27], *p* = 0.80 [0.57–4.75], *p* = 0.40
Baseline leukocytes	>10 × 10^9^/L vs ≤10 × 10^9^/L	1.98	[1.02–3.8], *p* = 0.04
Baseline CEA	>ULN vs normal	2.84	[0.93–8.70], *p* = 0.07
Two months CEA vs baseline CEA	Stable or increase vs decrease >50%	3.61	[1.68–7.75], *p* = 0.01
Baseline platelet	≥400 × 10^9^/L vs <400 × 10^9^/L	1.09	[0.55–2.14], *p* = 0.81

*CEA* carcinoembryonic antigen, *ULN* upper limit of normal.

### Factors associated with short duration of chemotherapy-free interval

Baseline characteristics of patients according to the CFI duration are presented in Supplementary Table S2. Univariate analysis revealed that male gender, WHO performance status of 1 or 2, unresected primary tumour, right colon primary, baseline leukocytes >10 × 10^9^/L, baseline platelet >400 × 10^9^/L, baseline alkaline phosphatase >300 IU/L, baseline CEA upper limit of normal, *BRAF* mutation and no tumour response at 2 months were associated with a short duration of CFI (Table 3). In multivariate analysis, male gender and no tumour response at 2 months were associated with a short CFI (Table 4). The multivariate analysis performed in the subgroup of patients with *BRAF* V600E mutation status available revealed that *BRAF*- mutated status was the only factor associated with a short CFI (OR = 4.59 [0.95; 22.26], *p* = 0.058).

**Table 3:** Univariate analysis of characteristics associated with a short duration (<3 months) of chemotherapy-free interval.

Characteristics		OR for progression	[95% CI], *p* value
Treatment arm	Maintenance vs observation	1.20	[0.81–1.77], *p* = 0.17
Gender	Male vs female	1.33	[0.88–2.02], *p* = 0.17
Age	≤65 vs >65	1.10	[0.74–1.62], *p* = 0.64
WHO performance status	1 vs 0 2 vs 0	1.35 7.10	[0.90–2.02], *p* = 0.09 [2.16–23.35], *p* = 0.001
Primary tumour resected	No vs yes	1.74	[1.16–2.60], *p* = 0.007
The number of metastatic sites	>1 vs 1	1.08	[0.72–1.60], *p* = 0.72
Primary location	Right colon vs left colon or rectum	1.49	[0.89–2.49], *p* = 0.13
Baseline leukocytes	>10 × 10^9^/L vs ≤10 × 10^9^/L	2.13	[1.32–3.44], *p* = 0.002
Baseline platelets	≥400 × 10^9^/L vs <400 × 10^9^/L	1.77	[1.06–2.95], *p* = 0.03
Baseline alkaline phosphatase	>300 vs ≤300	3.54	[2.04–6.13], *p* < 0.0001
Baseline CEA	>ULN vs normal	1.82	[1.02–3.22], *p* = 0.04
Two months CEA vs baseline CEA	Stable or increase vs decrease >50%	1.15	[0.64–2.11], *p* = 0.63
Tumour *KRAS*	Mutated vs wild type	1.06	[0.68–1.64], *p* = 0.81
Tumour *BRAF*	Mutated vs wild type	6.19	[1.74–22.05], *p* = 0.005
Tumour evaluation at 2 months	Stable disease vs complete or partial response	1.96	[1.29–2.99], *p* = 0.002
Tumour evaluation at 6 months	Stable disease vs complete or partial response	1.42	[0.92–2.19], *p* = 0.12

*CEA* carcinoembryonic antigen, *ULN* upper limit of normal.

**Table 4:** Multivariate analysis of characteristics associated with a short duration (< 3 months) of chemotherapy-free interval.

Characteristics	*N* = 172	OR for progression	[95% CI], *p* value
Gender	Male vs female	1.72	[0.92–3.25], *p* = 0.09
WHO performance status	1 vs 0 2 vs 0	1.33 3.38	[0.72–2.44], *p* = 0.36 [0.48–23.78], *p* = 0.22
Primary tumour resected	No vs yes	1.57	[0.84–2.98], *p* = 0.16
Primary location	Right colon vs left colon or rectum	1.35	[0.70–2.62], *p* = 0.37
Baseline leukocytes	>10 × 10^9^/L vs ≤10 × 10^9^/L	0.95	[0.43–2.08], *p* = 0.89
Baseline alkaline phosphatase	>300 vs ≤300 U/L	1.35	[0.54–3.37], *p* = 0.53
Baseline CEA	>ULN vs normal	1.94	[0.80–4.71], *p* = 0.14
Baseline platelet	≥400 × 10^9^/L vs <400 × 10^9^/L	1.32	[0.59–2.96], *p* = 0.50
Tumour evaluation at 2 months	Stable disease vs complete or partial response	1.90	[0.96–3.76], *p* = 0.07
Tumour evaluation at 6 months	Stable disease vs complete or partial response	1.33	[0.69–2.56], *p* = 0.39

*CEA* carcinoembryonic antigen, *ULN* upper limit of normal.

## Discussion

Our results showed that baseline-elevated leukocytes and stable or increasing CEA at 2 months were independent factors associated with progression during IC. In this study, we have investigated prognostic factors for early progression within the first 6 months of chemotherapy. In the PRODIGE 9 trial, the independent prognostic factors associated with a shorter PFS were PS 2 and *BRAF* mutation.^14^ *BRAF* mutation was also reported as a prognostic factor for shorter PFS in previous trials^10,15^ after both doublet and triplet chemotherapy combined with bevacizumab. Nevertheless, the *BRAF* mutation was not found as a prognostic factor of early progression in our study. Baseline CEA and early CEA variation during chemotherapy were already reported as associated with PFS or OS.^16,17^ Interestingly, the prognostic value of baseline CEA was reported in patients treated with FOLFIRI plus bevacizumab, but not with FOLFIRI plus cetuximab.^18^ In this study, the lack of decrease in CEA at 2 months was associated with an early progression, and potentially should be considered in order to intensify chemotherapy. Circulating DNA is described as another early marker of chemotherapy efficacy.^19^ Unfortunately, this biomarker was not collected in our study. CEA and circulating DNA monitoring have both advantages and limitations. CEA is easy to perform with low cost, but some tumours do not produce even CEA, and could not be evaluated with this marker. Circulating DNA requires specific technology, provides additional cost and needs further evaluation in a metastatic setting. It would be worthwhile to compare the predictive value of both markers. Elevated baseline leukocytes are prognostic of an early progression in this study. The Köhne criteria include this parameter as a prognostic factor for OS.^20^ However, elevated baseline leukocytes were not a prognostic factor for PFS on the main analysis of PRODIGE 9 trial.^14^ Thus, according to our results, both parameters, the lack of the CEA decrease level and the elevated baseline leukocytes as prognostic factors for early progression, should be confirmed in another series. Other prognostic factors for early progression could also be integrated as radiomic evaluation^21^ or biological markers beyond *BRAF* mutation as consensual molecular classification.^22^

Male gender and no tumour response at 2 months according to RECIST 1.1 criteria were found to be prognostic for a short first CFI. Previous studies have assessed prognostic factors for CFI or maintenance treatment. In the COIN trial that evaluated IC continuation compared with CFI, baseline thrombocytosis was associated with a short CFI.^23^ In our study, baseline thrombocytosis was associated with short CFI in univariate analysis but not in multivariate analysis, suggesting confounding factors or a lack of statistical power. In the CAIRO 3 trial, tumour response and synchronous metastasis were associated with a longer PFS in the maintenance arm with capecitabine plus bevacizumab.^12^ Patients with stable disease at the first evaluation have a shorter CFI, suggesting that CFI is not appropriate for those patients. In a pooled analysis of CAIRO 3 and AIO 0207 trials, female gender, synchronous-resected metastasis and *BRAF* mutation were associated with a longer OS if maintenance chemotherapy is performed compared with observation.^24^ It must be pointed out that in our study, *BRAF* mutation was the strongest negative predictor for CFI in univariate and in multivariate analysis in the subgroup of patients with *BRAF* status determination. In regard to these and our results, it seems reasonable to recommend a maintenance chemotherapy rather than CFI in patients with *BRAF*-mutated tumour.

Our study has several limitations. First, it is a post hoc unplanned analysis, a prospective trial comparing treatment adaptation according to prognostic factors with no adaptation, which could only demonstrate the validity of the concept. A switch to oxaliplatin and/or to anti-EGFR in the case of *RAS* wild-type tumour, or to other targeted therapies in the case of *BRAF* mutant tumour according to recent results,^25^ could be evaluated if the signal of early progression during induction is observed, and maintenance chemotherapy as 5FU or capecitabine plus bevacizumab combination could be compared with CFI if it was a signal of short CFI. In the subgroup of patients with tumour *BRAF* mutation, it would be of interest to compare continuous induction chemotherapy with capecitabine plus bevacizumab chemotherapy or encorafenib plus cetuximab. Second, all the patients received irinotecan and bevacizumab treatment; thus, whether our findings could be extended to first-line oxaliplatin and/or anti-EGFR-based chemotherapy remains to be demonstrated.

In conclusion, early progression may be anticipated in the case of elevated leukocytes at baseline, and no CEA decrease at first evaluation. Further study should be performed to evaluate other radiologic or biologic predictors. Caution should be taken before performing a CFI without maintenance treatment in patients with *BRAF*-mutated tumour or lack of tumour response at first evaluation. Our results would help making decisions for patients who prefer a complete CFI rather than maintenance chemotherapy. Further studies are needed in a larger number of patients to confirm these results and add eventually other prognostic factors.

## Supplementary information

Supplementary file

## Data Availability

Data supporting this publication are stored at the FFCD Data Center.
