# Supplementary material for: Predictive factors for early progression during induction chemotherapy and chemotherapy-free interval: analysis from PRODIGE 9 trial
Source: Br J Cancer. 2020 Feb 4;122(7):957–62. doi: 10.1038/s41416-020-0735-8 (PMC7109054; doi:10.1038/s41416-020-0735-8)
Supplement: Supplementary file 1 — Supplementary file [file 41416_2020_735_MOESM1_ESM.docx]

**Supplementary table 1: Baseline characteristics of patients with and without tumor progression during induction chemotherapy.**

|  | | **No tumor progression during IC** | **Tumor progression during IC** |
| --- | --- | --- | --- |
|  |  | (N=403) | (N=85) |
| **Age** | n | 403 | 85 |
|  | <= 65 years | 203 (50.4%) | 46 (54.1%) |
|  | >65 years | 200 (49.6%) | 39 (45.9%) |
| **Gender** | n | 403 | 85 |
|  | Male | 265 (65.8%) | 51 (60.0%) |
|  | Female | 138 (34.2%) | 34 (40.0%) |
| **WHO performance status** | n | 403 | 85 |
|  | 0 | 203 (50.4%) | 30 (35.3%) |
|  | 1 | 175 (43.4%) | 41 (48.2%) |
|  | 2 | 25 (6.2%) | 14 (16.5%) |
| **Köhne Criteria** | n | 403 | 85 |
|  | Low | 155 (38.5%) | 26 (30.6%) |
|  | Intermediate | 183 (45.4%) | 33 (38.8%) |
|  | High | 65 (16.1%) | 26 (30.6%) |
| **Primary tumor resected** | n | 402 | 85 |
|  | Yes | 236 (58.7%) | 45 (52.9%) |
|  | No | 166 (41.3%) | 40 (47.1%) |
| **Number of metastatic sites** | n | 403 | 85 |
|  | 1 | 161 (40.0%) | 31 (36.5%) |
|  | >1 | 242 (60.0%) | 54 (63.5%) |
| **Primary location** | n | 390 | 85 |
|  | Colon | 300 (76.9%) | 70 (82.4%) |
|  | Colon and Rectum | 5 (1.3%) | 1 (1.2%) |
|  | Rectum | 85 (21.8%) | 14 (16.5%) |
| **Localization** |  | 210 | 49 |
|  | Left colon | 128 (61.0%) | 29 (59.2%) |
|  | Left and right colon | 1 (0.5%) | 1 (2.0%) |
|  | Right Colon | 81 (38.6%) | 19 (38.8%) |
| **Tumor *KRAS*** | n | 313 | 62 |
|  | Wild-Type | 170 (54.3%) | 32 (51.6%) |
|  | Mutated | 143 (45.7%) | 30 (48.4%) |
| **Tumor *BRAF*** | n | 283 | 52 |
|  | Wild-Type | 266 (94.0%) | 48 (92.3%) |
|  | Mutated | 17 (6.0%) | 4 (7.7%) |

IC: Induction chemotherapy

**Supplementary Table 2: Baseline characteristics according the duration of the 1^st^ chemotherapy free-interval**

|  | | CFI <3 months | CFI from 3 to 5 months | CFI >5 months |
| --- | --- | --- | --- | --- |
|  |  | (N=128) | (N=100) | (N=116) |
| **Age** | n | 128 | 100 | 116 |
|  | <= 65 years | 65 (50.8%) | 58 (58.0%) | 55 (47.4%) |
|  | >65 years | 63 (49.2%) | 42 (42.0%) | 61 (52.6%) |
| **Gender** | n | 128 | 100 | 116 |
|  | Male | 89 (69.5%) | 71 (71.0%) | 71 (61.2%) |
|  | Female | 39 (30.5%) | 29 (29.0%) | 45 (38.8%) |
| **WHO performance status** | n | 128 | 100 | 116 |
|  | 0 | 58 (45.3%) | 53 (53.0%) | 69 (59.5%) |
|  | 1 | 58 (45.3%) | 43 (43.0%) | 47 (40.5%) |
|  | 2 | 12 (9.4%) | 4 (4.0%) | 0 (0.0) |
| **Köhne Criteria** | n | 128 | 100 | 116 |
|  | Low | 49 (38.3%) | 34 (34.0%) | 52 (44.8%) |
|  | Intermediate | 48 (37.5%) | 52 (52.0%) | 58 (50.0%) |
|  | High | 31 (24.2%) | 14 (14.0%) | 6 (5.2%) |
| **Primary tumor resected** | n | 127 | 100 | 116 |
|  | Yes | 66 (52.0%) | 60 (60.0%) | 80 (69.0%) |
|  | No | 61 (48.0%) | 40 (40.0%) | 36 (31.0%) |
| **Primary location** | n | 124 | 97 | 110 |
|  | Colon | 101 (81.5%) | 75 (77.3%) | 78 (70.9%) |
|  | Colon and rectum | 0 (0.0) | 3 (3.1%) | 1 (0.9%) |
|  | Rectum | 23 (18.5%) | 19 (19.6%) | 31 (28.2%) |
| **Localization** |  | 73 | 49 | 61 |
|  | Left colon | 40 (54.8%) | 35 (71.4%) | 38 (62.3%) |
|  | Left and Right colon | 0 (0.0) | 1 (2.0%) | 0 (0.0) |
|  | Right colon | 33 (45.2%) | 13 (26.5%) | 23 (37.7%) |
| **Number of metastatic sites** | n | 128 | 100 | 116 |
|  | 1 | 54 (42.2%) | 34 (34.0%) | 52 (44.8%) |
|  | >1 | 74 (57.8%) | 66 (66.0%) | 64 (55.2%) |
| **Tumor *KRAS*** | n | 96 | 82 | 91 |
|  | Wild-Type | 55 (57.3%) | 39 (47.6%) | 54 (59.3%) |
|  | Mutated | 41 (42.7%) | 43 (52.4%) | 37 (40.7%) |
| **Tumor *BRAF*** | n | 84 | 75 | 83 |
|  | Wild-Type | 74 (88.1%) | 74 (98.7%) | 81 (97.6%) |
|  | Mutated | 10 (11.9%) | 1 (1.3%) | 2 (2.4%) |

CFI: Chemotherapy free-interval

**List of Investigators/Collaborators**

Dr Véronique Guérin-Meyer (Centre Paul Papin, Angers) ; Dr Benjamin Linot (Centre Paul Papin, Angers) ; Dr François-Xavier Caroli-Bosc (Centre Hospitalier Universitaire Hôtel Dieu, Angers), Dr Caroline Couffon (Centre Hospitalier Universitaire Hôtel Dieu, Angers) ; Dr Dominique Luet (Centre Hospitalier Universitaire Hôtel Dieu, Angers) ; Dr Guillaume Roquin (Centre Hospitalier Universitaire Hôtel Dieu, Angers) ; Dr Anne-Laure Villing (Centre Hospitalier, Auxerre) ; Dr Dominique Sevin-Robiche (Clinique Sainte Marguerite, Auxerre) ; Dr Franck Audemar (Centre Hospitalier de la Côte Basque, Bayonne) ; Dr Anne Cadier-Lagnes (Centre Hospitalier de la Côte Basque, Bayonne), Dr Anne-Claire Dupont-Gossart (Centre Hospitalier Universitaire Jean Minjoz, Besançon) ; Dr Mohamed Ramdani (Centre Hospitalier, Béziers) ; Dr Yann Le Bricquir (Centre Hospitalier, Béziers) ; Dr Dany Gargot (Centre Hospitalier, Blois) ; Pr Thomas Aparicio (Centre Hospitalier Universitaire Avicenne, Bobigny) ; Dr Yves Becouarn (Institut Bergonié, Bordeaux) ; Dr Dominique Bechade (Institut Bergonié, Bordeaux) ; Dr Vincent Bourgeois (Centre Hospitalier Duchenne, Boulogne sur Mer) ; Dr Corinne Sarda (Centre Hospitalier, Castres) ; Dr Nathalie Hess-Laurens (Centre Hospitalier, Castres) ; Dr Jean-Marie Vantelon (Centre Leonard de Vinci, Dechy) ; Dr Claire Giraud (Centre Leonard de Vinci, Dechy) ; Dr François Ghiringhelli (Centre Georges-François Leclerc, Dijon) ; Dr Véronique Lorgis (Centre Georges-François Leclerc, Dijon) ; Dr Julie Vincent (Centre Georges-François Leclerc, Dijon) ; Dr Jean-Louis Jouve (Centre Hospitalier le Bocage, Dijon) ; Pr Laurent Bedenne (Centre Hospitalier le Bocage, Dijon) ; Dr Paul-Arthur Haineaux (Centre Hospitalier le Bocage, Dijon) ; Pr Côme Lepage (Centre Hospitalier le Bocage, Dijon) ; Dr Geneviève Boilleau-Jolimoy (Centre de Radiothérapie du Parc, Dijon) ; Dr Anne-Marie Queuniet (Centre Hospitalier, Elbeuf) ; Dr Stéfanie Oddou-Lagraniere (Centre Hospitalier Chicas, Gap) ; Dr François Dewaele (Centre Hospitalier les Oudairies, La Roche-sur-Yon) ; Dr Thierry Chatellier (Centre Hospitalier les Oudairies, La Roche-sur-Yon) ; Dr Roger Faroux (Centre Hospitalier les Oudairies, La Roche-sur-Yon) ; Dr Guillaume Medinger (Centre Hospitalier les Oudairies, La Roche-sur-Yon) ; Dr Anne Thirot-Bidault (Centre Hospitalier Universitaire Bicêtre, Le Kremlin Bicêtre), Dr Oana Cojocarasu (Centre Hospitalier, Le Mans) ; Dr Florence Kikolski (Centre Hospitalier Robert Boulin, Libourne) ; Dr Philippe Martin (Centre Bourgogne, Lille) ; Dr Dominique Genet (Clinique François Chénieux, Limoges) ; Dr Joël Ezenfis (Centre Hospitalier, Longjumeau) ; Dr Joëlle Egreteau (Centre Hospitalier Bretagne Sud, Lorient) ; Dr Isabelle Cumin (Centre Hospitalier Bretagne Sud, Lorient) ; Dr Régine Lamy (Centre Hospitalier Bretagne Sud, Lorient) ; Dr Julien Forestier (Hôpital Edouard Herriot, Lyon) ; Dr Catherine Lombard-Bohas (Hôpital Edouard Herriot, Lyon) ; Dr Yann Molin (Hôpital Edouard Herriot, Lyon) ; Dr Thomas Walter (Hôpital Edouard Herriot, Lyon) ; Dr Christelle De La Fouchardière (Centre Léon Bérard, Lyon), Dr Françoise Desseigne (Centre Léon Bérard, Lyon), Dr Denis Peré-Vergé (Centre Hospitalier Saint Joseph, Lyon) ; Dr Jean-Christophe Souquet (Centre Hospitalier Universitaire de La Croix Rousse, Lyon) ; Dr Lionel Wander (Centre Hospitalier Universitaire de La Croix Rousse, Lyon) ; Dr Yves Rinaldi (Hôpital Européen, Marseille) , Dr Nicolas Barrière (Hôpital Européen, Marseille) ; Dr Mohamed Gasmi (Hôpital Nord, Marseille) ; Dr Muriel Duluc (Centre Hospitalier Universitaire La Timone, Marseille) ; Pr Jean-François Seitz (Centre Hospitalier Universitaire La Timone, Marseille) ; Dr Emmanuelle Norguet-Monnereau (Centre Hospitalier Universitaire La Timone, Marseille) ; Pr Laétitia Dahan (Centre Hospitalier Universitaire La Timone, Marseille) ; Dr Christophe Locher (Centre Hospitalier, Meaux) ; Dr Hatem Salloum (Centre Hospitalier, Meaux); Dr Farah Zerouala-Boussaha (Centre Hospitalier, Meaux) ; Dr Patrick Texereau (Centre Hospitalier Layné, Mont de Marsan) ; Dr Jérôme Dauba (Hôpital Layné, Mont de Marsan) ; Dr Miguel Carreiro (Centre Hospitalier, Montauban) ; Dr Nathalie Gérardin (Centre Hospitalier, Montauban); Dr Ahmed Azzedine (Centre Hospitalier, Montélimar) ; Dr Stéphane Jacquot (Institut du cancer du Val d’Aurelle, Montpellier) ; Dr Catherine Becht (Institut du cancer du Val d’Aurelle, Montpellier); Dr Eric Janssen (Institut du cancer du Val d’Aurelle, Montpellier); Dr Lafforgue Béatrice (Institut du cancer du Val d’Aurelle, Montpellier) ; Dr Eric François (Centre Antoine Lacassagne, Nice) ; Dr Philippe Follana (Centre Antoine Lacassagne, Nice) ; Dr Abakar Mahamat (Centre Hospitalier Universitaire l’Archet, Nice) ; Dr Suzanne Nguyen, Centre Hospitalier, Beauvais) ; Dr Jacques Cretin (Clinique Valdegour, Nîmes) ; Dr Jérôme Meunier (Centre Hospitalier de la Source, Orléans) ; Dr Corina Cornila (Centre Hospitalier de la Source, Orléans) ; Dr Gaël Goujon (Centre Hospitalier Universitaire Bichat, Paris) ; Dr Bruno Landi (Hôpital Européen Georges Pompidou, Paris) ; Pr Julien Taieb (Hôpital Européen Georges Pompidou, Paris) ; Pr Philippe Rougier (Hôpital Européen Georges Pompidou, Paris) ; Dr Isabelle Trouilloud (Hôpital Européen Georges Pompidou, Paris) ; Dr Aziz Zaanan (Hôpital Européen Georges Pompidou, Paris) ; Dr Céline Lepère (Hôpital Européen Georges Pompidou, Paris) ; Pr Jean-Baptiste Bachet (Centre Hospitalier Pitié Salpêtrière, Paris) ; Dr Touraj Mansourbakht (Centre Hospitalier Pitié Salpêtrière, Paris) ; Dr Jean-Marc Gornet (Centre Hospitalier Universitaire Saint-Louis, Paris) ; Dr Laurent Cany (Polyclinique Francheville, Périgueux) ; Dr Charles-Briac Levaché (Polyclinique Francheville, Périgueux) ; Dr Faiza Khemissa-Akouz (Centre Hospitalier Saint Jean, Perpignan) ; Dr Sophie Pesque-Penaud (Centre Hospitalier Universitaire Haut-Lévêque, Pessac) ; Dr Eric Terrebonne (Centre Hospitalier Universitaire Haut-Lévêque, Pessac) ; Dr Julien Vergniol (Centre Hospitalier Universitaire Haut-Lévêque, Pessac) ; Dr Pierre-Luc Etienne (Centre Cario, Plérin-Sur-Mer) ; Dr Mathieu Baconnier (Centre Hospitalier Annecy Genevois, Pringy) ; Dr Emmanuel Maillard (Centre Hospitalier Annecy Genevois, Pringy) ; Dr Laetitia Stefani (Centre Hospitalier Annecy Genevois, Pringy) ; Pr Olivier Bouché (Centre Hospitalier Universitaire Robert Debré) ; Dr Julien Volet (Centre Hospitalier Universitaire Robert Debré) ; Dr Marie-Claude Gouttebel (Centre Hospitalier Drôme Nord, Romans-sur-Isère) ; Dr Marc Porneuf (Centre Hospitalier Yves Le Foll, Saint-Brieuc) ; Dr Vincent Quentin (Centre Hospitalier Yves Le Foll, Saint-Brieuc) ; Pr Jean-Marc Phelip (Hôpital Bellevue, Saint-Etienne) ; Dr Xavier Coulaud (Hôpital Bellevue, Saint-Etienne) ; Dr Jaafar Bennouna (Institut de Cancérologie de l’Ouest, Saint-Herblain) ; Dr Hélène Senellart (Institut de Cancérologie de l’Ouest, Saint-Herblain) ; Sandrine Hiret (Institut de Cancérologie de l’Ouest, Saint-Herblain) ; Pr Jean-Yves Douillard (Institut de Cancérologie de l’Ouest, Saint-Herblain) ; Dr Hélène Castanie (Institut de Cancérologie de l’Ouest, Saint-Herblain) ; Dr Laurent Gasnault (Centre Joliot Curie, Saint Martin Boulogne) ; Dr Thierry Chatellier (Clinique Mutualiste de l'Estuaire, Saint Nazaire) ; Dr Ivan Graber (Clinique Trenel, Saint Colombe les Vienne) ; Dr Annunziato Alessio (Clinique Trenel, Saint Colombe les Vienne) ; Dr Meher Ben Abdelghani (Centre Paul Strauss, Strasbourg) ; Dr Christian Borel (Centre Paul Strauss, Strasbourg), Dr Christine Belletier (Centre Paul Strauss, Strasbourg) ; Dr Etienne Suc (Clinique Saint Jean du Languedoc, Toulouse) ; Pr Thierry Lecomte (Centre Hospitalier Trousseau, Tours) ; Pr Etienne Dorval-Danquechin (Centre Hospitalier Trousseau, Tours) ; Dr Zied Ladhib (Centre Hospitalier, Valence) ; Dr Marie-Christine Kaminsky (Institut de Cancérologie de Lorraine, Vandoeuvre Les Nancy) ; Pr Thierry Conroy (Institut de Cancérologie de Lorraine, Vandoeuvre Les Nancy) ; Dr Lynn Rob (Institut de Cancérologie de Lorraine, Vandoeuvre Les Nancy) ; Dr Valérie Boige (Institut Gustave Roussy, Villejuif) ; Dr David Malka (Institut Gustave Roussy, Villejuif) ; Dr Pascal Burtin (Institut Gustave Roussy, Villejuif) ; Dr Elie Zrihen (Institut Gustave Roussy, Villejuif) ; Pr Michel Ducreux (Institut Gustave Roussy, Villejuif) ; Dr Antoine Hollebecque (Institut Gustave Roussy, Villejuif)
